# Outcome of vaginal mesh reconstructive surgery in multiparous compared with grand multiparous women: Retrospective long-term follow-up

**DOI:** 10.1371/journal.pone.0176666

**Published:** 2017-05-04

**Authors:** Gil Levy, Yoav Peled, Anat From, Irena Fainberg, Sarit Barak, Amir Aviram, Haim Krissi

**Affiliations:** 1 Division of Female Pelvic Medicine, Department of Obstetrics and Gynecology, Mayanei HaYeshua Medical Center, Bnei Brak, Israel; 2 Urogynecology Unit, Department of Obstetrics and Gynecology, Helen Schneider Hospital for Women, Rabin Medical Center – Beilinson Hospital; affiliated with Sackler Faculty of Medicine, Tel Aviv University, Tel Aviv, Israel; UNITED STATES

## Abstract

We aimed to compare the long-term surgical outcome and complications of multiparous and grand multiparous women undergoing reconstructive surgery with vaginal mesh implants for repair of pelvic organ prolapse. This retrospective, long-term follow-up (28.17±20.7 months) comprised 113 women who underwent surgical reconstructive surgery with vaginal polypropylene mesh in a high parity rate population medical center. The women were divided into 2 groups (multiparous and grand multiparous) and each group was evaluated for objective and subjective surgical outcome. Patient demographics and surgical data were retrieved from electronic medical records. Outcome measure included POP-Q exam as objective outcome and validated Pelvic Floor Distress Inventory questionnaire (PFDI) to assess subjective outcome. Average age of patients was 62±7.9 (range 42–83) years. Average parity was 5.6±3.1 (range 1–14). There were 54 (47.7%) multiparous women and 59 (52.3%) grand multiparous women. The grand multiparous women were younger than the multiparous women and had a significantly higher degree of prolapse. At the last follow-up, the only significant difference was related to symptoms of an overactive bladder. In conclusion, long-term follow-up demonstrates that vaginal mesh surgery in grand multiparous women offers anatomical and subjective cure rates comparable to multiparous women.

## Introduction

Pelvic organ prolapse (POP) negatively affects the quality of life of millions of women worldwide [1]. The lifetime prevalence is 3–6% when defined by symptoms, and up to 50% when based upon vaginal examination [2]. Prevalence increases with age, instrumental deliveries, body mass index (BMI) and parity [3]. The lifetime risk for undergoing surgery for definitive treatment is up to 19% [4]. The vaginal route for surgery is associated with decreased cost and shorter operating time, reduced hospital stay and earlier return to daily activities compared with the abdominal approach [5].

Vaginal delivery is considered the proven risk factor for POP. Each added vaginal delivery results in added trauma to the pelvic floor connective tissue and an increased degree of POP [6]. Higher parity women have extensive pelvic damage and will present with third- and fourth-degree POP when compared to lower parity counterparts [7–9].

Since the FDA warnings regarding the use of mesh material for vaginal reconstructive surgery in 2011 [10], there has been a decline in the number of mesh surgeries and increasing number of reports describing comparable results between native tissue repair and mesh surgery. Most of the recent published data includes patients with an average parity of <4 with second- and third-degree prolapse. Data on outcome of surgical treatment with vaginal mesh in patients with extensive pelvic damage or high parity is scarce.

Our medical center (Mayanei Hayeshua Hospital) serves an orthodox Jewish population with a high average parity per woman. Secondary to the high parity, the percentage of women with symptomatic prolapse is higher, as is the percentage of third- and fourth-degree prolapses. In order to evaluate and compare the outcome of mesh surgery in multiparous and grand multiparous women in our community, we reviewed our patient population by anatomical and subjective long-term results.

## Materials and methods

We conducted a retrospective cohort study of all women who underwent POP vaginal surgery using a polypropylene mesh from 2009 to 2013. The study was approved by our local Institutional Review Board: Helsinki Committee of Maynei Hayeshua Medical center.

The IRB committee waived the need for separate content form for each participant.

Information regarding prolapse, urinary, bowel, and sexual symptoms was accessed, according to the appropriate standardized International Continence Society (ICS) definitions. Each woman underwent a routine physical examination and site-specific vaginal examination in the lithotomy position with a Sim’s speculum, during a maximal Valsalva maneuver. Each vaginal compartment was evaluated for defects in pelvic support. All measurements and staging were performed according to the ICS scoring system for pelvic organ prolapse quantification (POP-Q) [11].

Data on demographic parameters, age, BMI, parity, co-morbidities, smoking, previous hysterectomy and preoperative POP-Q score were collected from the electronic medical records. Patients were contacted by telephone and asked to attend the clinic to review possible complications. Each participant underwent a POP-Q examination and was asked to complete a Pelvic Floor Distress Inventory (PFDI) validated questionnaire.

### Statistical analysis

Statistical analysis was performed using the SPSS software (IBM SPSS statistics version 23.0, Armonk, NY, USA). Comparison between continuous variables was performed with the Student’s t test for normally distributed variables, and the Mann-Whitney U test for non-Gaussian distributed variables. Categorical variables were compared using the Chi-square test, and pre- and post-procedure paired variables were compared using the Wilcoxon signed-rank test.

## Results

Overall, 138 women underwent vaginal reconstructive surgery using polypropylene mesh between 2009–2013. One hundred thirteen women (81.9%) completed the follow-up, which was an average of 28±20.7 months (Median = 29, IQR = 38). Average age of the study population was 62.0±7.9 (range 42–83) years, and BMI measured 27.33 (range 18–41). Average parity was 5.6±3.7 (range 1–14) with only 2 instrumental deliveries. Thirteen women (11.5%) had cesarean deliveries from which only two had second cesarean delivery. Three patients (2.6%) had significant intraoperative bleeding (>500 cc), but did not require blood transfusion; laparotomy was performed in one case (0.9%) to control the bleeding. Nineteen women (16.8%) had post-surgical erosions including both symptomatic and asymptomatic findings, from which 3 (2.6%) underwent surgical resection. All other erosions were treated successfully with local estrogen therapy. Eight women (7%) complained of dyspareunia at the follow-up visit and 5 (4.4%) women complained of pelvic pain. No organ perforation or significant symptomatic hematomas were documented. Overall, there was a significant improvement in all POP-Q points comparing before and after measurements (Table 1).

**Table 1:** POP-Q* for the whole group before and after the procedure and by group prior to surgery.

Variable	Before Median (range)			After Median (range)
Aa	3.0 (-3 - +3)			-3.0 (-3 - +3)
Ba	3.0 (-3 - +8)			-3.0 (-3 - +6)
C	1.0 (-8 - +8)			-7.0 (-9 - +6)
GH	5.0 (2–8)			4.5 (3–7)
PB	2.0 (2–5)			3.0 (2–5)
TVL	8.0 (6–10)			8.0 (6–9)
Ap	-1.0 (-3 - +3)			-3.0 (-3 - +3)
Bp	-1.0 (-3 - +8)			-3.0 (-3 - +6)
D	-4.0 (-8 - +8)			-7.0 (-9 - +6)
	GM	M	p value	
Aa	3.0 (-3 - +3)	2.5 (-3 - +3)	**0.05**	
Ba	4.0 (-3 - +8)	2.5 (-3 - +8)	**0.003**	
C	1.0 (-8 - +8)	-0.5 (-8 - +8)	**0.003**	
GH	6.0 (2–7)	4.0 (2–8)	**<0.001**	
PB	2.0 (2–3)	2.0 (2–5)	**0.004**	
TVL	8.0 (7–10)	8.0 (6–9)	0.07	
Ap	-1.0 (-3 - +3)	-1.0 (-3 - +3)	0.09	
Bp	0.0 (-3 - +8)	-1.0 (-3 - +8)	**0.03**	
D	-3.0 (-8–8)	-5.0 (-7 - +8)	**0.003**	

* Bump RC et al. [11].

Ba, maximum descent of anterior vaginal wall; Bp, maximum descent of posterior vaginal wall; C, maximum descent of the cervix or vaginal cuff; POP-Q, pelvic organ prolapse quantification; TVL, total vaginal length

The study population was divided by parity into two groups: women with 5 and more deliveries [grand multiparas (GM)] formed the study group (GM) and women with 1–4 deliveries [multiparas (M)] acted as the control group. Demographic characteristics of both groups are detailed in Table 2. The M group was younger then the GM group. The number of previous cesarean deliveries was higher in the GM group, which can be rationalized statistically. The POP-Q score of the GM group was significantly higher than the M group with the exception of the Total vaginal length (TVL) and Ap measurements.

**Table 2:** Patient's characteristics.

Variable	GM group (n = 59)	M group (n = 54)	*P* value
Age (years), mean±SD	60.4±7.0	63.7±8.6	**0.03**
Body mass index (kg/m2), mean±SD	27.2±4.8	27.3±3.3	0.89
Gravidity, median (range)	8.0 (5–14)	3.0 (1–4)	**<0.001**
Previous Cesarean Delivery, n (%)	10 (17.2)	2 (3.7)	0.02
Previous Operative Vaginal Delivery, n (%)	5 (8.5)	6 (11.1)	0.64
Previous prolapse procedure, n (%)	0 (0)	1 (1.0)	0.80
Fecal incontinence, n (%)	1 (1.7)	2 (3.7)	0.51
Constipation, n (%)	7 (11.9)	6 (11.1)	0.90
Hormone replacement therapy, n (%)	0 (0)	2 (3.7)	0.14
Previous hysterectomy, n (%)	5 (8.5)	10 (18.5)	0.12
Diabetes mellitus, n (%)	5 (8.5)	5 (9.3)	0.88
Chronic hypertension, n (%)	12 (20.7)	13 (24.1)	0.67
Smoking, n (%)	1 (1.7)	2 (3.7)	0.51

GM, grand multipara; M, multipara; POP-Q, pelvic organ prolapse quantification

All values presented are means±SD or number (%).

Table 3 summarizes the surgical procedures performed and details the type of mesh used for the surgery. The GM group had significantly more sacrospinous ligament fixation (SSLF) procedures than the M group. Also the number of hysterectomies was significantly higher in the GM group than the M group. All other surgical procedures were comparable between the study and control group.

**Table 3:** Surgical procedures.

Variable, n(%)	GM group (n = 59)	M group (n = 54)	*P* value
SSLF	26 (44.1)	7 (13.0)	<0.001
TVTO	29 (49.2)	22 (40.7)	0.37
SI TVT	7 (11.9)	3 (5.6)	0.24
SI general	36 (61.0)	25 (46.3)	0.12
Vaginal hysterectomy	24 (40.7)	12 (22.2)	0.04
Post mesh GAC	2 (3.4)	1 (1.9)	0.61
Post mesh Endofast	1 (1.7)	0 (0)	0.34
Post mesh Elevate	1 (1.7)	0 (0)	0.34
Post mesh prolift	6 (10.2)	10 (18.5)	0.20
Post mesh General	10 (16.9)	11 (20.4)	0.64
Ant. mesh GAC	2 (3.4)	0 (0)	0.17
Ant. mesh Endofast	1 (1.7)	0 (0)	0.34
Ant. mesh Elevate	3 (5.1)	3 (5.6)	0.91
Ant. mesh prolift	45 (76.3)	40 (74.1)	0.79
Ant. mesh General	51 (86.4)	43 (79.6)	0.33

GM, grand multipara; M, multipara

SSLF = sacrospinous ligament fixation, TVTO^®^ = Johnson and Johnson product, TVT^®^ = Johnson and Johnson product, GAC = Graft augmented colporrhaphy using self-cut Gynamesh^®^ anchored proximally to the SSLF bilaterally using prolene sutures and distally to the para-urethral tissue bilaterally. Elevate^®^—AMS product, Prolift^®^—Johnson and Johnson product, Endofast^®^–IBI product. General = summary of all mesh procedures per compartment.

In our institute, we routinely consult women prior to surgery regarding the option of hysterectomy as part of the prolapse repair procedure. Women who opt for hysterectomy are advised that the hysterectomy does not have an impact on the success rate of the prolapse repair. Women with no risk factors for uterine malignancy and with no history of postmenopausal uterine pathologic finding are encouraged to perform the surgical repair without hysterectomy.

Table 4 summarizes the results of the follow-up. The anatomical results presented in the POP-Q score were comparable between both groups. The PFDI scores for high satisfaction rate were also comparable between the groups. All the postoperative complications showed no significant differences. During the follow-up examination, all mesh erosions (symptomatic and asymptomatic) were documented.

**Table 4:** Follow-up results.

Variable	GM group (n = 59)	M group (n = 54)	*P* value
Follow-up duration (month)	35.0 (37.0)	22.5 (36.0)	0.3
POP-Q* after the procedure			
Aa	-3.0 (-3 - +3)	-3.0 (-3 - +3)	0.57
Ba	-3.0 (-3 - +6)	-3.0 (-3 - +2)	0.13
C	-7.0 (-9 - +6)	-7.0 (-9 - +3)	0.15
GH	5.0 (3–7)	4.0 (3–5)	0.12
PB	3.0 (2–4)	3.0 (2–5)	0.55
TVL	8.0 (6–9)	8.0 (6–9)	0.84
Ap	-3.0 (-3 - +3)	-3.0 (-3 - +3)	0.96
Bp	-3.0 (-3 - +6)	-3.0 (-3 - +4)	0.44
D	-7.0 (-9 - +6)	-7.0 (-8 - +6)	0.41
PFDI	15.0 (0–56)	17.0 (0–47)	0.65
Postoperative complications/additional surgery			
Local estrogen treatment	2 (3.4)	2 (3.7)	0.93
Posterior repair	1 (1.7)	0 (0)	0.34
Erosion remove	2 (3.3)	1 (1.9)	0.29
Additional Sacrocolpopexy	2 (3.4)	0 (0)	0.17
Vaginal hysterectomy and posterior and anterior colporrhaphy	6 (10.2)	3 (5.6)	0.37
Stress urinary incontinence	2 (3.4)	2 (3.7)	0.93
Voiding difficulty	0 (0)	1 (1.9)	0.29
Overactive bladder	23 (39.0)	9 (16.7)	0.009
Fecal symptoms	1 (1.7)	2 (3.7)	0.51
Chronic pelvic pain/dyspareunia	3 (5.1)	2 (3.7)	0.72
Recurrent posterior wall prolapse	2 (3.4)	1 (1.9)	0.61
Recurrent vault prolapse	2 (3.4)	0 (0)	0.17
Recurrent Uterine prolapse	7 (11.9)	4 (7.4)	0.43
Recurrence of any site prolapse	11 (18.6)	5 (9.3)	0.15
Urinary tract infection	1 (1.7)	0 (0)	0.34
Local infection	3 (5.1)	4 (7.4)	0.34
Dyspareunia	5 (8.5)	8(14.8)	0.29
Mesh exposure	8 (13.6)	11 (20.4)	0.33
Bleeding (>500ml)	3 (5.1)	0 (0)	0.09
Hematoma	0 (0)	0 (0)	NA

* Bump RC et al. [11].

GM, grand multipara; M, multipara; PFDI, Pelvic Floor Distress Inventory

Complications included the need for second surgery for rectocele repair, removal of mesh erosion, abdominal sacrocolpopexy for failure of the first procedure and hysterectomy with anterior repair.

Prolapse recurrence of was detailed by compartment: rectocele, vault and uterine prolapse, with an overall of 5 patients (9.3%) in the M group and 11 patients (18.6%) in the GM group. No significant difference in overall prolapse recurrence was calculated between the groups (*p* = 0.15).

In order to evaluate the impact of surgical outcome in both groups, we compared the pre- and post-surgery POP-Q points in each group (Tables 5 and 6) and found significant improvements in all points. Impact of surgery on the preoperative prolapse status was calculated with an improvement factor for each POP-Q point (i.e., improvement Aa = post op Aa—pre op Aa). Negative results denoted better anatomical results. Table 7 compares the improvement factor in each group. The only significant improvement difference was calculated at the GH and PB points. All other points improved statistically in both groups equally.

**Table 5:** POP Q* for multiparous women before and after the procedure.

POP Q	M group (n = 54)		*P* value
Variable	Before	After	
Aa	2.5 (-3 - +3)	-3.0 (-3 - +3)	<0.001
Ba	2.5 (-3 - +8)	-3.0 (-3 - +2)	<0.001
C	-0.5 (-8 - +8)	-7.0 (-9 - +3)	<0.001
GH	4.0 (2–8)	4.0 (3–5)	0.03
PB	2.0 (2–5)	3.0 (2–5)	0.05
TVL	8.0 (6–9)	8.0 (6–9)	0.02
Ap	-1.0 (-3 - +3)	-3.0 (-3 - +3)	<0.001
Bp	-1.0 (-3 - +8)	-3.0 (-3 - +4)	<0.001
D	-5.0 (-7 - +8)	-7.0 (-8 - +6)	0.01

* Bump RC et al. [11].

M, multipara;

**Table 6:** POP Q* for grand multiparous women, before and after the procedure.

	GM group (n = 59)	
Variable	Before	After
Aa	3.0 (-3 - +3)	-3.0 (-3 - +3)
Ba	4.0 (-3 - +8)	-3.0 (-3 - +6)
C	1.0 (-8 - +8)	-7.0 (-9 - +6)
GH	6.0 (2–7)	5.0 (3–7)
PB	2.0 (2–3)	3.0 (2–4)
TVL	8.0 (7–10)	8.0 (6–9)
Ap	-1.0 (-3 - +3)	-3.0 (-3 - +3)
Bp	0.0 (-3 - +8)	-3.0 (-3 - +6)
D	-3.0 (-8 - +8)	-7.0 (-9 - +6)

* Bump RC et al. [11].

GM, Grandmultipara;

**Table 7:** Improvement of POP Q*, by parity.

Variable	GM (n = 59)	M group (n = 54)	*P* value
Aa	-5.0 (-6 - +6)	-5.0 (-6 - +6)	0.22
Ba	-5.0 (-11 - +5)	-5.0 (-11 - +5)	0.22
C	-6.5 (-16 - +5)	-5.0 (-14 - +8)	0.42
GH	-1.0 (-3 - +1)	0.0 (-3 - +2)	0.03
PB	1.0 (0–2)	0.0 (-2 - +3)	0.05
TVL	-1.0 (-3 - +1)	0.0 (-3 - +3)	0.14
Ap	-2.0 (-6 - +5)	-1.0 (-6 - +3)	0.15
Bp	-2.0 (-11 - +3)	-1.0 (-9 - +4)	0.09
D	-2.5 (-16 - +1)	-2.0 (-4 - +1)	0.19

* Bump RC et al. [11].

GM, grand multipara; M, multipara

## Discussion

Our study found similar results in terms of cure rate and complications in POP reconstructive surgery with mesh interposition in GM versus M women.

This study was conducted in a medical center that serves a community with high average parity per woman. Prenatal care and women’s health centers, as well as contraceptive techniques are available in the community. Our medical center also has the lowest cesarean delivery rate in the country (9.8%), which probably adds to higher rate of obstetrical trauma in the community. These factors explains the high rate of POP and the high percentage of third- and fourth-degree prolapse compared to other low parity communities. The higher level of pelvic trauma secondary to high parity was documented in our study with significant differences in the POP-Q measurements when comparing both groups. Anterior compartment POP-Q measurements were significantly higher in the GM group compared to the M group. The TVL measurement, which represents passive length and does not express damage to the pelvic support system, did not differ between the groups. The same was true for the Ap points but we were unable to explain this result. The GM women were significantly younger then the M women at the time of surgery. POP become symptomatic at an earlier age in women with higher parity which can explain the age discrepancy between the groups.

As expected, the pre-operative POP-Q score of the GM group was significantly higher than the M group with the exception of the TVL and Ap measurements. The posterior wall exception can be explained by the difference between the impact of birth trauma to the posterior compartment compare to the impact of birth trauma to the anterior compartment.

The GM group had significantly more sacrospinous ligament fixation (SSLF) procedures than the M group. This probably represents the need to address the apical prolapse in the GM group compared to the M group.

The GM group had significantly more vaginal hysterectomies.

We consider the vaginal mesh implant as a level 2 prolapse reinforcement rather than a combined level 1 and 2 treatment. Adding the SSLF is considered a level 1 treatment in our institution, specifically since some of the mesh kits (i.e., Ant. Prolift^®^) are anchored at the “pre-ischial spine” anchoring points rather than to the sacrospinous ligament itself. To evaluate the impact of SSLF on our results, we re-analyzed the data without the SSLF cases and did not find a significant difference regarding failures or measurements of POP-Q points C or TVL. A similar analysis was conducted to evaluate the impact of vaginal hysterectomy on the results, and again, no significant differences were calculated.

In the present study, the overall outcome of surgical mesh use is comparable to previously published data [12]. The overall anatomical failure was calculated at 18.6% in the GM group, which was higher than the 9.3% in the M group. Although this difference is not significant, the tendency can still be explained by the advanced pelvic trauma in the GM group [13].

The intra- and postoperative complications are comparable to previously published data with only three surgical cases with intra-operative bleeding exceeding 500 cc. Mesh erosions [14,15] and dyspareunia [16–18] were within the range of other reports in the literature [19].

Our findings show that there is no difference in the clinical outcome between the M and the GM group. These results suggest that the mesh implant has the ability to provide similar benefit in cases with a higher degree of prolapse and advanced level of pelvic floor damage presented in the GM group. These results were confirmed also by analyzing the POP-Q measurements per each group, separately (Tables 5 and 6) and by the improvement analysis shown in Table 7. We were unable to explain the higher number of postoperative overactive bladder in the GM group.

Using a validated PFDI Hebrew questionnaire [20] we were able to evaluate the subjective surgical outcome. The overall PFDI results revealed high satisfaction rate which is similar to previously reported rates [21].

The overall satisfaction rate was high in both groups, with an equal correlation between the subjective and objective satisfaction rate in both groups. We consider the subjective results to be the most important in the evaluation of therapy for quality-of-life issue as in POP.

### Strengths and limitations

This study is limited by its retrospective design and the relatively small sample size. The lack of PFDI results prior to surgery for comparison also limits the study results. However, this unique population of grand multiparous women with advanced obstetric pelvic trauma adds important clinical information in our understanding of mesh usage in pelvic floor surgery.

### Conclusion

Our data suggest that the use of vaginal mesh in surgical correction of third- and fourth-degree POP has the same long-term outcome in grand-multiparous patients as in multiparous patients. Further studies are needed to understand the advantage of mesh use compared to native tissue repair in the GM group.

## Supporting information

S1 Dataset S1_levy et al dataset. (XLSX)

## Abbreviations

BMI: body mass index
ICS: International Continence Society
POP: pelvic organ prolapse
SSLF: sacrospinous ligament fixation
